# Quality and Quantity Assessment of the Water Repellent Properties of Functional Clothing Materials after Washing

**DOI:** 10.3390/ma15113825

**Published:** 2022-05-27

**Authors:** Mateusz Kowalski, Renata Salerno-Kochan, Irena Kamińska, Małgorzata Cieślak

**Affiliations:** 1 Department of Non-Food Product Quality and Safety, Cracow University of Economics, Rakowicka 27, 31-510 Cracow, Poland; salernor@uek.krakow.pl; 2 Department of Chemical Textile Technologies, Łukasiewicz Research Network—Textile Research Institute, Brzezińska 5/15, 92-103 Lodz, Poland; irena.kaminska@iw.lukasiewicz.gov.pl (I.K.); malgorzata.cieslak@iw.lukasiewicz.gov.pl (M.C.)

**Keywords:** hydrophobic textiles, spray test, water repellency, surface properties

## Abstract

The aim of the research was to evaluate the changes in the surface properties of five functional clothing materials with water-repellent finishes (including PFC-free finish) after 1, 5, and 10 washes with three detergents. A new approach to the interpretation of the water-repellent properties of textile materials is presented, based on two techniques, i.e., the spray test method and contact angle measurements. The results showed that washing materials with hydrophobic finishes can cause significant changes in their properties, which are mainly dependent on the composition and structure of the material, as well as the type of hydrophobic finish. The PFC-free finish is the least resistant to washing. For all materials with PFC finishes, the water repellency depends on the fluorine content on the surface and fabric topography. It was also found that increasing washing frequency resulted in a gradual decrease in water repellency. The loss of water repellency below an acceptable level (Grade 3) occurred after the fifth washing for all materials. Significant differences in the interpretation of the results of the spray test and contact angle measurements were observed. Using these methods separately provides information on the changes in the surface properties of the tested materials; however, their parallel application allows for obtaining complementary data, which is important for the proper interpretation of results.

## 1. Introduction

One of the most important properties of the multifunctional textiles used in outerwear is water repellency. Along with resistance to water penetration (waterproofness) and water vapour permeability (breathability), it has a direct impact on the wearer’s comfort [1,2]. It is responsible for protecting against water soaking into the fabric, which, as demonstrated by Rengasamy [3], can lead to reduced breathability and deterioration of the garment’s thermal properties, causing an acceleration of the heat transfer process and, consequently, heat stress and severe discomfort for the garment wearer [4]. According to Lomax [5], wet clothing in contact with the skin can cause up to 25 times faster heat loss by conduction; so, the proper protection of outerwear against the effects of precipitation water is essential for providing the wearer with the desired physiological comfort.

Water repellency properties in textiles can be achieved in a number of ways, using hydrophobic fibers, dense fabric structure, and chemical and/or physical surface modifications [6,7]. In functional outerwear made of multilayer materials, the most widely used finishes are durable water repellents (DWR), whose role is to lower the surface free energy of the material and achieve water-repellent properties. The low surface energy of the material, which is lower than the surface tension of water, causes droplets to undergo a pearling phenomenon and be “repelled” from the surface [8,9]. Starting in the mid-20th century, the most widely used finishes with hydrophobic properties were fluorine-containing compounds that impart easy-care properties to materials, such as water and oil repellency, stain repellency, and soil and stain-release properties. In addition, fluorine-based finishes are characterized by high chemical and thermal stability and durability [9,10]. However, hydrophobic finishes based on fluorinated compounds, particularly with “long” perfluoroalkyl chains, exhibit negative environmental impacts, due to the presence of strong carbon–fluorine bonds that resist biodegradation [11,12,13]. Alternative compounds are increasingly being used to impart hydrophobic properties to materials, including short-chain SFPs or non-fluorinated finishes (PFC-free finishes), which include paraffins, silicones, dendrimer chemicals, and nanoparticles [14,15,16,17]. However, as was shown by the results of Schellenberger et al. [10], Chowdhury et al. [18], and Davies [19], hydrophobic finishes devoid of fluorocarbon compounds have mostly lower durability, compared to fluorocarbon finishes. The study conducted by W.L. Gore and associates [20] shows that a lower durability of PFC-free finishes requires a more frequent renewal of the garment’s water-repellent properties, making it likely that jackets with PFC-free and fluorocarbon-based finishes, considering their full life cycle, may have similar environmental impacts.

The effectiveness and durability of water-repellent finishes during use depends on their type. According to Davies [21], the most important factors that reduce their effectiveness include mechanical impacts (e.g., friction and bending), prolonged exposure to water, and maintenance processes. The influence of maintenance treatments on the reduction of the durability of hydrophobic finishes was also confirmed by Gargoubi et al. [11], Schellenberger et al. [12], Kowalski et al. [22], Solomon [23], Yurdakul et al. [24], Shi et al. [25], Deng et al. [26], and Ivanova and Zaretskaya [27], who observed that the deterioration of water-repellent properties of materials with hydrophobic finishes increases with the number of washing cycles. Additionally, it was shown that the durability of water-repellent properties of materials depends on the type of laundry detergent used [28,29].

In the referenced works, the water repellency of the materials was evaluated mainly using the spray test method and contact angle measurement, which are the most commonly used techniques for evaluating water-repellent properties. Depending on the method adopted for testing the water repellency of materials, the conclusions drawn from the results obtained may differ significantly. Solomon [23], Yurdakul et al. [24], Kowalski and Salerno-Kochan [28], and Brooks [30], who evaluated the durability of hydrophobic finishes using the spray test method, showed a significant decrease in the water-repellent properties of textiles after washing processes. In contrast, Shi et al. [25], Deng et al. [26], and Ivanova and Zaretskaya [27], who used the contact angle measurement method to evaluate the hydrophobic properties of materials after washing processes, showed a high durability of the tested hydrophobic finishes. In a study presented by Deng et al. [26], even after 50 washes, the contact angle values determined for a cotton fabric with a hydrophobic finish exceeded 150°. In the studies conducted so far, the contact angle was determined only for water. The use of standard liquids with different values of the surface free energy components allows one to determine the value of the surface free energy, thus indicating the direction and reasons of changes in the surface properties of materials.

The aim of this work addresses the following issues:

- The evaluation of changes in the surface properties of clothing materials with declared water repellency using the spray test method and method of measurement of contact angles with standard liquids, combined with the determination of the value of surface free energy and its dispersion and polar components;
- The evaluation of the influence of the material characteristics on the changes of water repellency properties after washing;
- The evaluation of the influence of washing conditions on the changes of water repellency properties.

## 2. Materials and Methods

### 2.1. Textile Materials

The subject of the study was five multilayer clothing materials with water-repellent, windproof, waterproof, and vapor-permeable properties, consisting of woven fabrics made of polyester (PES), recycled polyester (rPES), or polyamide 6.6 (PA 6.6) laminated with hydrophilic or microporous membranes made of polyester (PES), polyurethane (PU), or polytetrafluoroethylene (PTFE), with additional hydrophilic polyurethane coating. In addition to the composition of the outer and membrane layers, the materials also differed in their structural parameters and the type of finish that imparts water-repellent properties, with the type of hydrophobic finish not precisely specified, which presented an additional research challenge.

The materials were sourced from three different manufacturers in Germany, Japan, and the United States, which produce the membranes and membrane laminates used in functional outerwear, among other applications. Five materials were selected to be the “flagship” products of each manufacturer. The research material was selected on the basis of literature data and the analysis of market products, so they were the types of layered structures most commonly used in rainproof outerwear.

Table 1 shows the characteristics of the materials, based on the manufacturers’ information.

Table 2 shows the characteristics of the structural parameters of the materials made on the basis of own research.

**Figure 1:**
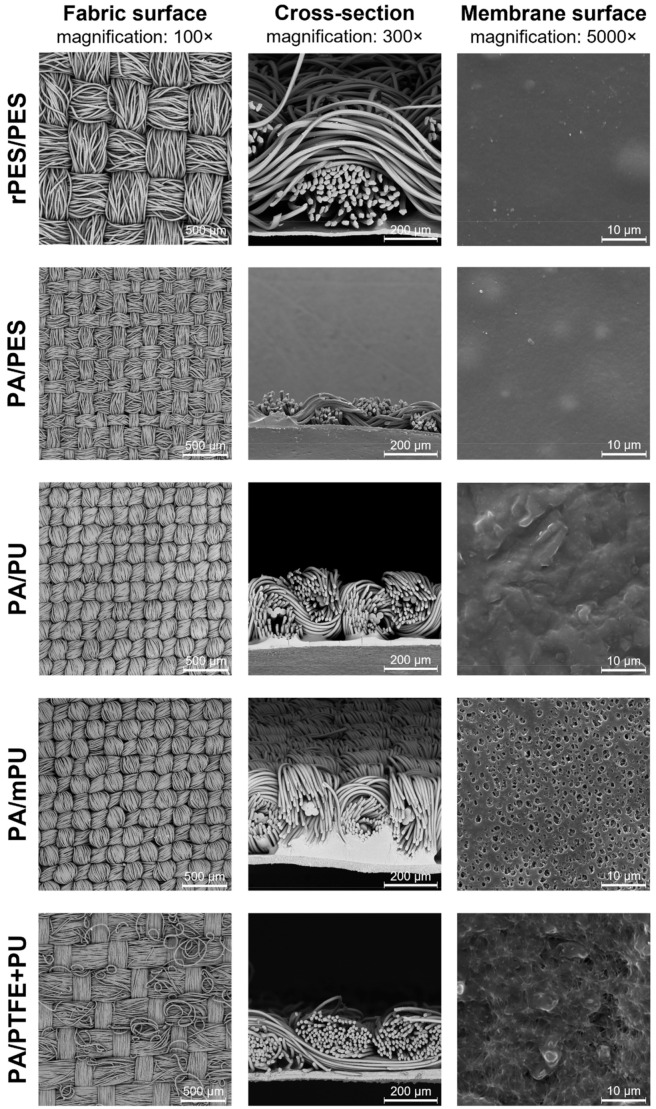
SEM images of fabric surfaces (100×), cross-sections (300×), and membrane surfaces (5000×) of studied materials.

Figure 1 presents SEM images of fabric surfaces (magnified 100×), cross-sections (magnified 300×), and membrane surfaces (magnified 5000×) of the studied materials. The SEM images of materials were made using a scanning electron microscope VEGA 3 (TESCAN, Brno, Czech Republic), equipped with a secondary electron detector. Images were taken in a high-vacuum mode at 30 kV.

### 2.2. Detergents and Washing Procedures

Three types of washing agents were used in the study. Detergent D1 was a non-phosphate powder reference detergent (A) without an optical brightening agent (James Heal, Halifax, UK). Its composition is standardized and specified in the Annex K of ISO 6330. Detergent D2 was a liquid detergent dedicated to multifunctional clothing materials with water-repellent, waterproof, and breathable properties (Nikwax, Wadhurst, UK, approx. EUR 2.0/1 wash). It contained polycarboxylates (<5%), soap (5–15%), and nonionic surfactants. Detergent D3 was a universal, concentrated liquid for washing coloured products (Procter & Gamble, Cincinnati, OH, USA, approx. EUR 0.2/1 wash). It included anionic surfactants (5–15%), nonionic surfactants (<5%), phosphonates, soap, enzymes, benzisothiazolinone, methylisothiazolinone, fragrances, alpha-isomethyl ionone, citronellol, and geraniol.

Three variants of washing were used in the study. In variant V1, materials were washed according to ISO 6330 (method 4N), using detergent 1 and washing machine Vascator FOM71 MP LAB (type A). In variants V2 and V3, the Whirlpool TDLR 60220 washing machine, with top loading and a horizontal axis drum, was used. Samples were washed using a gentle wash program (without prewash, temperature: 40 °C, spin speed: 800 rpm, and total time: 45 min., including washing, rinsing, and spinning). In variant V2, dedicated liquid detergent D2 for washing waterproof and breathable fabrics was used. In variant V3, universal concentrated liquid detergent D3 was used. For a single wash, according to the standard requirements and recommendations of the detergent manufacturer, 20 g of detergent D1, 150 mL of detergent D2, or 75 mL of detergent D3 were added. A total of 1, 5, and 10 washes of all materials were performed for each variant. After the washing treatment for each variant, the samples were dried by hanging. Table 3 shows the designations of samples used in the study.

### 2.3. EDS Analysis of Water Repellency Finishes Composition 

Qualitative and quantitative elemental composition analyses were performed to identify the types of water-repellent finishes. The tests were performed on the face side (fabric with hydrophobic finish). 

The analyses were performed on an INCA Energy EDS X-ray microanalyzer from Oxford Instruments Analytical (Oxford, UK), coupled with a VEGA3 electron microscope from TESCAN (Brno, Czech Republic), equipped with a monocrystalline lithium (Li)-activated silicon detector, enabling the detection of elements from beryllium (Be) to uranium (U), with a maximum resolution of 128 eV for the Mn (K_α_) line. X-ray microanalysis of the surface of fabrics with hydrophobic finishes was carried out under low-vacuum, at a pressure of 30 Pa, using an electron beam energy of 20 kV, without spraying the samples with a conductive substance, at a magnification of 300×. Qualitative and quantitative analyses of the chemical composition were carried out from areas of 0.5 mm^2^ in triplicate. The values of percentages of elements were determined by INCA program. The results of weight percentages of individual elements are given with an accuracy of 0.01%.

### 2.4. Spray Test for Water Repellency Analysis

**Figure 2:**
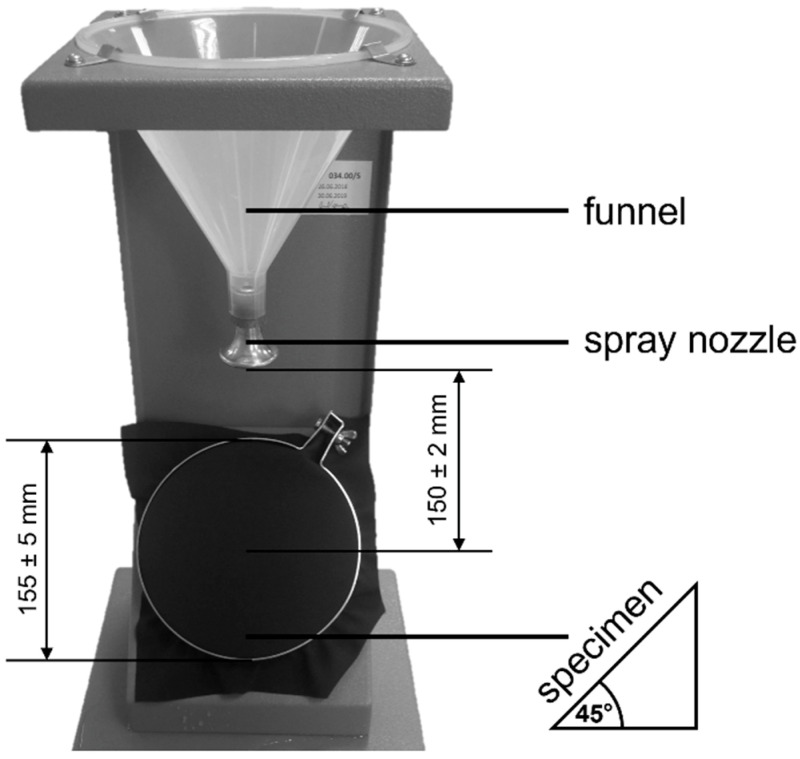
Spray tester used in the study.

The resistance to surface wetting measurements were made before and after washing using the Spray Tester M232 (SDL International, Sunnyvale, CA, USA), in accordance with ISO 4920. Figure 2 shows the spray tester used in the resistance to surface wetting tests.

ISO 4920 recommends the use of a five-grade scale of photographic standards for resistance to surface wetting (1–5); however, during this study, we decided to use the six-grade scale specified in AATCC TM22, which introduced an additional grade “0”, differentiating between “complete wetting of the entire specimen face beyond the spray points” and “complete wetting of the entire face of the specimen”. Table 4 provides verbal descriptions of each degree of the surface wetting resistance scale.

The tests of water repellency were carried out in three repetitions for each material, assessing the surface of each specimen on a scale from 0 to 5. Due to great difficulty in assessing the degree of surface wetting of some specimens, which often did not change significantly after the washing processes, it was decided to use intermediate grades in the assessment. Because of the adopted deviation from ISO 4920 standard, the degree of resistance to surface wetting of the material was determined as the mean value from three measurements, and it was quoted with an accuracy of 0.1 degree.

### 2.5. Goniometric Analysis of Surface Properties

**Figure 3:**
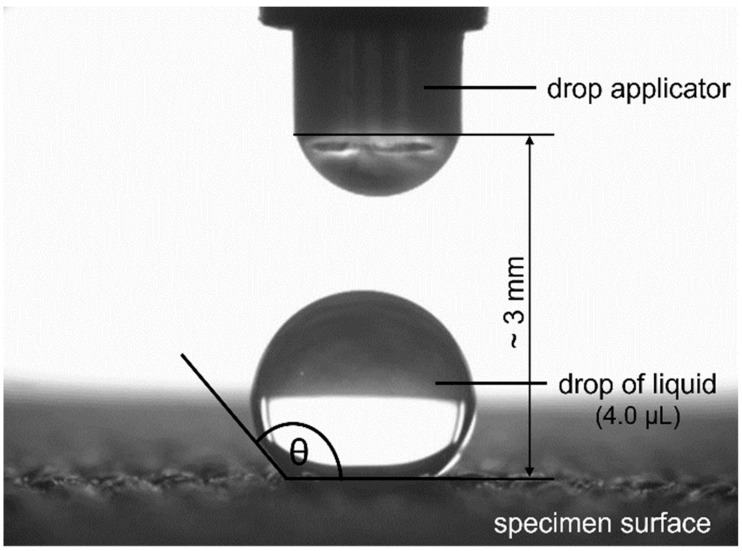
Measurement of contact angle.

The analyses of the surface properties of tested fabrics were carried out by goniometric method, using a goniometer PGX (Fibro System AB, Stockholm, Sweden). Figure 3 presents the principle of measuring the contact angle (θ) using a goniometer.

Two standard liquids with known surface tensions and different values of dispersive and polar components were applied (Table 5). A drop of liquid, with a volume of 4 μL, was applied. Five repetitions for each sample were made. The measurements were carried out at a temperature of 21 ± 1 °C, and the relative air humidity was 40 ± 2%.

The surface free energy (γ_S_) was calculated using the Owens–Wendt method, as the sum of dispersive (γ^d^) and polar (γ^p^) components (Equation (1)). The dispersive component (γ^d^) determines the value of London forces (resulting from dipole and induced dipole interactions). The polar component (γ^p^) is understood as the sum of the polar, hydrogen, inductive, and acid-base interactions. To calculate the free surface energy using the Owens–Wendt method, a system of two linear equations with two unknowns (γ_S_^d^ and γ_S_^p^) is created, based on Equation (2) [31,32]:

γ_S_ = γ_S_^d^ + γ_S_^p^ (1)

γ_l_ (1 + cosθ) = 2[(γ_l_^d^γ_S_^d^)^1/2^ + (γ_l_^p^γ_S_^p^)^1/2^] (2)

where γ_S_—surface free energy of solid, γ_S_^d^ and γ_S_^p^—dispersion and polar components of solid, γ_l_—surface free energy of liquid, γ_l_^d^ and γ_l_^p^—dispersion and polar components of liquid, and θ—contact angle.

### 2.6. Statistical Analysis

Statistical significance of the results (significance level of *p* < 0.05) was determined for samples after washes, in comparison with the reference sample, by the data analysis software system Statistica (version 13.3, TIBCO Software Inc., Palo Alto, CA, USA), using Student’s *t*-test for dependent samples and analysis of variance (ANOVA). To compare the results obtained with the different test methods, the data were analyzed by correlation analysis calculating the Pearson correlation coefficient.

## 3. Results and Discussion

### 3.1. Compositions of Water Repellent Finishes 

Table 6 presents the results of the EDS analysis of the elemental composition of the outer surfaces of the fabrics of the studied materials, which enabled the identification of the chemical elements used in the water-repellent finishes. 

**Figure 4:**
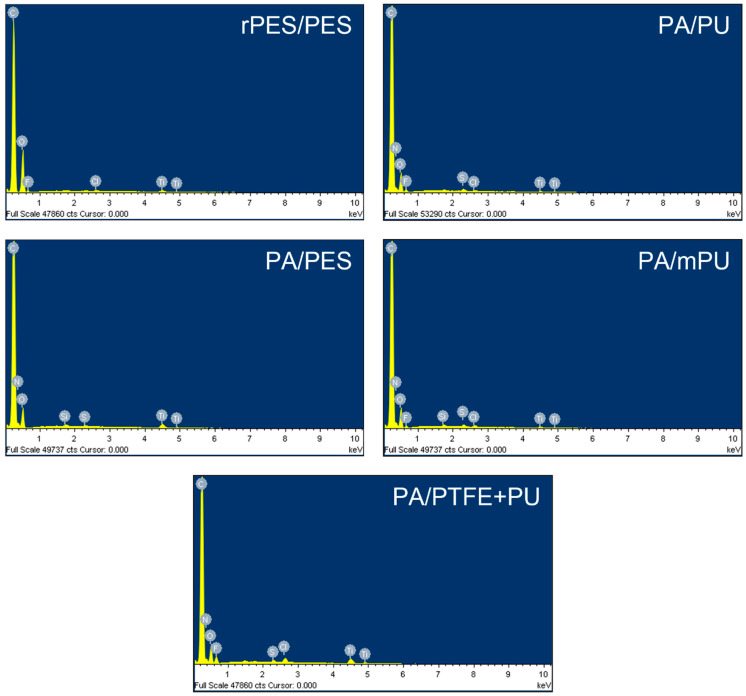
The sample EDS spectra of the fabric surfaces.

Figure 4 presents the sample EDS spectra of the fabric surfaces.

The presence of the elements with the highest percentage of carbon (C), oxygen (O), and nitrogen (N) is due to the structure of the fibrous polymers (C and O—polyester fabric; C, O, and N—polyamide fabric). Fluorine (F), derived from the hydrophobic finish, was identified on the surfaces of the four fabrics. The highest percentage of fluorine was on the PA/PTFE + PU surface (5.13 ± 0.36%). On the surface of PA/PU (2.31 ± 0.03%) and rPES/PES (2.15 ± 0.33%), it was more than twice smaller; on PA/mPU (1.49 ± 0.13%), it was more than three times smaller. No fluorine was detected on the PA/PES surface, but silicon (Si) was present. This was confirmed by another type of hydrophobic finish, the so-called PFC-free (Table 1). The presence of titanium was also detected on the surface of all fabrics (most on PA/PTFE + PU).

### 3.2. Water Repellency Results

**Figure 5:**
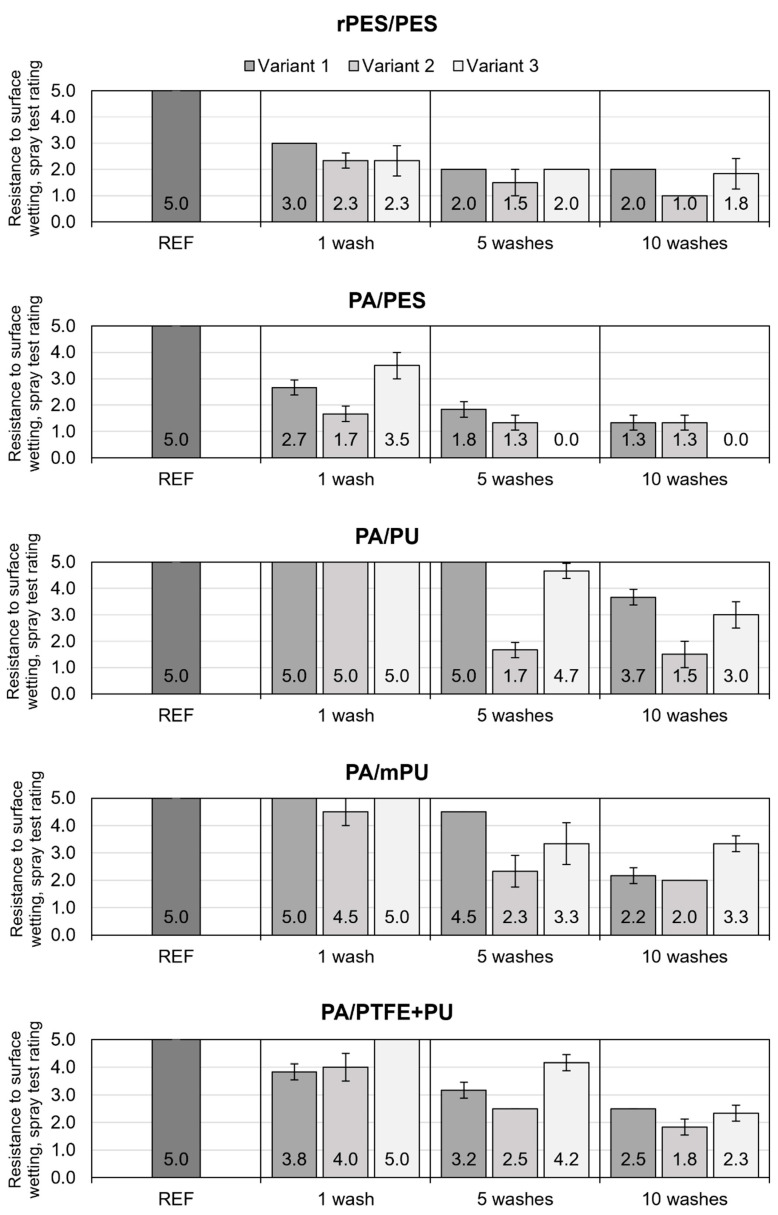
Resistance to surface wetting (spray test) results.

Figure 5 presents the mean values of the surface water repellency, as evaluated by the spray test method and determined before and after 1, 5, and 10 washes, according to all variants.

All materials showed very good water repellency before washing. No traces of wetting were observed on the surfaces of the samples, so all materials were granted the maximum level of water repellency—5.0. After washing, a decrease in water resistant properties was observed, which varied depending on the type of detergent and washing frequency (Figure 5). Based on the averaged values of the measurements for all the materials, after 1, 5, and 10 washes, the mean decrease in water repellency was determined for each of the three variants. It ranged from 0.8 degrees after the first washing (according to the variant V3) to 3.5 degrees after ten washes (according to the variant V2). In all analyzed variants and washing times, the changes in the values of water repellency were significantly different from the initial values (*p* < 0.001).

The highest resistance to surface wetting was recorded for the PA/PU material, retaining full water repellency after one wash in all three variants (V1/1, V2/1, and V3/1), as well as in the V1/5 variant, followed by the PA/mPU (V1/1 and V3/1) and PA/PTFE + PU (V3/1) materials. The PA/PU and PA/mPU materials are characterized by both the high linear density of the weft and warp and hydrophobic finish, based on fluorine compounds, whose content, related to the number of carbon–fluorine bonds, significantly influences the increase in water repellency. A significant effect of the carbon chain length of a fluorocarbon compound on the effectiveness of water-repellent finishes was demonstrated by Audenaert et al. [33], who evaluated the degree of water repellency of materials with fluorine-based hydrophobic finishes of different chemical structures.

The absence of such a finish, in the case of PA/PES material, although it had the highest linear density of weft and warp, but also the lowest thickness (Table 2), contributed to a significant decrease in water repellency after the first wash (Figure 5). These observations are consistent with the findings of Schellenberger et al. [10], Chowdhury et al. [18], and Davies [19], who showed that hydrophobic finishes devoid of fluorocarbon compounds have lower efficiency and durability, compared to fluorocarbon finishes.

However, as our own research has shown, the mere presence of fluorocarbon finishes does not guarantee the durability of the water-repellent properties in washing processes. This is evidenced by a marked decrease in the water repellency of the rPES/PES material, despite the fact that, on its surface, a fluorine content comparable to that of the PA/PU material was identified, i.e., 2.15 ± 0.33% and 2.31 ± 0.03%, respectively. The reason for the decrease in the durability of the water-repellent properties of this material may be found in the different raw material composition of the surface fabric (polyester), as well as in its structure, which was characterized by the lowest linear densities of weft and warp (Table 2 and Figure 1). When the hydrophobic finish is damaged, this structure may promote the penetration of water droplets into the inter-fiber spaces.

All materials, after five washing cycles, according to variant V2, obtained a water resistance rating below grade 3, which, in the literature [34,35,36], is given as the minimum acceptable grade that materials with hydrophobic properties should have after washing treatments. For variants V1 and V3, values below 3 were achieved by rPES/PES and PA/PES materials after the fifth washing. After the tenth washing, the acceptable value of water repellency was retained by the PA/PU (according to V1 and V3) and PA/mPU (according to V3) materials washed.

In order to evaluate the influence of the washing variant on the durability of the water-repellent properties of the materials, the differences between the mean values for all materials after washing, according to each of the three variants and the initial values, were determined. A positive value for the difference was interpreted as an increase in the value of the parameter, while a negative value was interpreted as its decrease, in comparison with the REF samples. The values of the differences determined for each washing variant were then subjected to analysis of variance (ANOVA), in order to determine whether statistically significant correlations existed between the compared groups, corresponding to the washing variants. The results of the analysis are presented in Table 7.

The analysis did not show a statistically significant effect of the washing variant on the durability of the water-repellent properties of the materials subjected to the washing processes (*p* = 0.064). The smallest changes (decrease) in water repellency, in comparison with the REF sample, were observed after washing according to variant V3 (mean ± SD: −1.94 ± 1.59; median: −1.83). The greatest changes were observed after washing according to variant V2, the conditions of which, including the type of detergent used (D2), caused a marked decrease in water repellency (mean ± SD: −2.76 ± 1.18; median: −3.25). This result contradicts the D2 detergent manufacturer’s declaration, according to which, the use of a specialised detergent does not contribute to the deterioration of the garment’s water-repellent properties and should even “renew” the existing water-repellent finish (DWR). According to the manufacturer, only after 5–6 washing cycles may the garment lose its water repellency, which would require a treatment to “renew” the water-repellent properties.

### 3.3. Goniometric Analysis

Table 8 summarizes the values of the contact angle for the fabric side of the materials before and after washings for water (θ_W_) and diiodomethane (θ_DIM_). 

All materials exhibited good hydrophobic properties, both before washing (REF) and after all washing cycles, as evidenced by the high values of θ_W_, which were greater than 90°. In the literature [21,37,38], this value is referred to as “limiting”, differentiating between hydrophilic (θ < 90°) and hydrophobic (θ > 90°) surfaces. For the diiodomethane wetting angle measurements, the initial values for all materials also exceeded 90°. The highest angle values, θ_W_ and θ_DIM_, before washing were recorded for rPES/PES materials, which is due to both fabric composition (PES) and surface topography (Figure 1). At the same percentage of fluorine content as for the PA/PU material, the value of θ_W_ was higher by 6.7°. The above observations highlight the importance of the influence of both the physical structure and chemical nature of the textile substrate on its water repellency, as demonstrated in the study presented by Song et al. [39].

**Figure 6:**
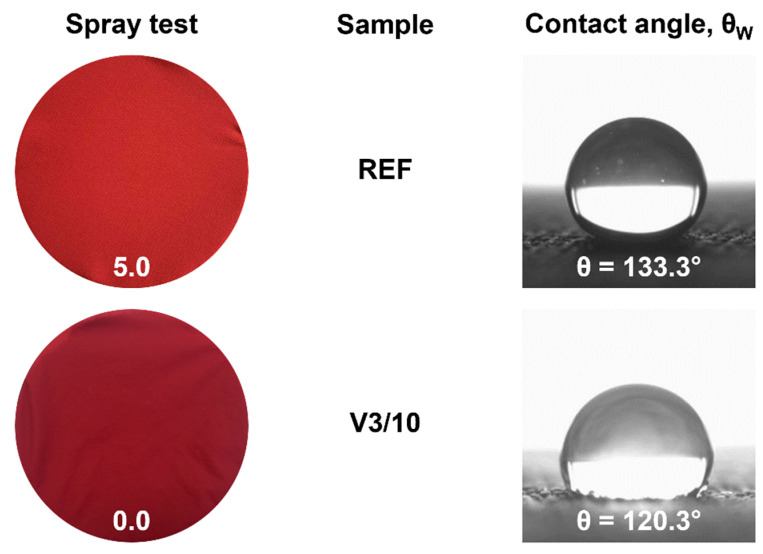
The sample results of spray test and contact angle measurements (θ_W_) for PA/PES fabric.

After washing, in most of the analyzed cases, there was a decrease in the mean values of θ_W_. The lowest values of θ_W_ were determined for the material with a PFC-free hydrophobic finish (PA/PES), after 5 and 10 washing cycles in the universal washing agent (V3). The determined values of the angle θ_W_ were 12.5° (−7.3%) and 14.6° (−10.8%) lower, respectively, compared to the REF sample, but they still remained high (above 120°), even after 10 washing cycles (Table 8). The PA/PES material, for which the greatest reduction in mean angle θ_W_ values was observed at the same stages of the spray test (V3/5 and V3/10), obtained the grade of complete wetting of the entire face (grade 0). Figure 6 presents the surface appearance of the PA/PES fabric after the spray test and contact angle measurement results for the REF samples and V3 variant after 10 washing cycles (V3/10).

The explanation for these differences in interpretation is that the spray test and goniometric methods provide different information, due to the different nature of the tests. During the spray test, the material, placed at a 45° angle, is subjected to a short, but intense (dynamic), impact of 250 mL of rainwater from a height of 150 mm. The high-volume water droplets hit the surface of the fabric, where the water runs down, reflecting the actual conditions that the fabric may be exposed to during rainfall. In the goniometric method of measuring the contact angle, a single drop of liquid, with a volume of 4 µL, was applied in static conditions. The contact angle values were influenced by physico-chemical interactions, mainly at the liquid–solid and liquid–air interfaces [31,40,41]. Using standard liquids differing in the values of polar and dispersion components and surface tension, it is possible to determine the surface free energy and components of the material under study, which is an important source of information regarding the physico-chemical properties of its surface and susceptibility to adsorption and adhesion.

For the PA/PES material, the greatest decrease in average θ_DIM_ values below 90° (V3/10), and even below 50° (V1/1, V2/1, V2/10), was also observed. This may be due to the fabric structure (Figure 1). The smallest thickness of the fabric, resulting from the use of thin yarns, and clearances seen in the microscopic images (incomplete filling of weft and warp) cause structural irregularities. This fabric also has a different hydrophobic finish (Table 6). Large local changes can occur after washing. In the case of diiodomethane, whose specific gravity is more than three times that of water for the same droplet volume, the diiodomethane droplet penetrates the clearances more easily, which can lead to an underestimation of the wetting angle. Residual detergent on the surface of the material can also contribute to this. 

The magnitude and nature of the changes in the mean values of θ_W_ and θ_DIM_ after washing also depended on the number of washing cycles performed and type of detergent used. Table 9 shows the results of the significance analysis of the differences between the initial values of the contact angles, θ_W_ and θ_DIM_, and surface free energy, as well as its components, with the values determined after 1, 5, and 10 washings, according to three variants (N = 20, because the calculations took the results of five measurements for four materials into account). The results for the PA/PES material with PFC-free finish were omitted from the analysis, due to the different types of finish and unusual contact angle gradients (Table 8).

In the case of contact angle measurements for water, with the exception of the V3/1 variant (*p* = 0.081), in which, for the PA/PU, PA/mPU, and PA/PTFE + PU materials, there was a slight increase in the angle θ_W_ (Table 8), there was a statistically significant (*p* < 0.05) decrease in mean values of θ_W_ (Table 9). For variant V1, it amounted, on average, to −3.5%, with −3.0% for variant V2 and −1.8% for variant V3. In each of the three variants, the average decrease in the wetting angle value, except for the mentioned case (V3/1), was the greater the higher the number of times of washing. In comparison with the REF sample, after the first washing, there was, on average, a 0.6% decrease in the value of the angle θ_W_; after five washings, it was less than 3% (2.7%), and, after ten washings, it was almost 5% (4.9%). The greatest decrease in the average θ_W_ values was observed after 10 washing cycles, according to variant V2, i.e., with the use of a special detergent. The values of the contact angle were lower than the initial ones by 7.1°, on average (−5.3%) (Table 9), and the highest decrease was observed for the PA/PTFE + PU material, for which, the determined value of angle θ_W_ was 12.5° (−9.2%) lower, compared to the REF sample (Table 8). As can be seen from the analysis presented in Table 9, the differences in the values of the contact angle for diiodomethane were statistically significant for only the variants V2/1 and V2/10; moreover, in both these cases, they were significantly lower, in comparison with the REF sample.

**Figure 7:**
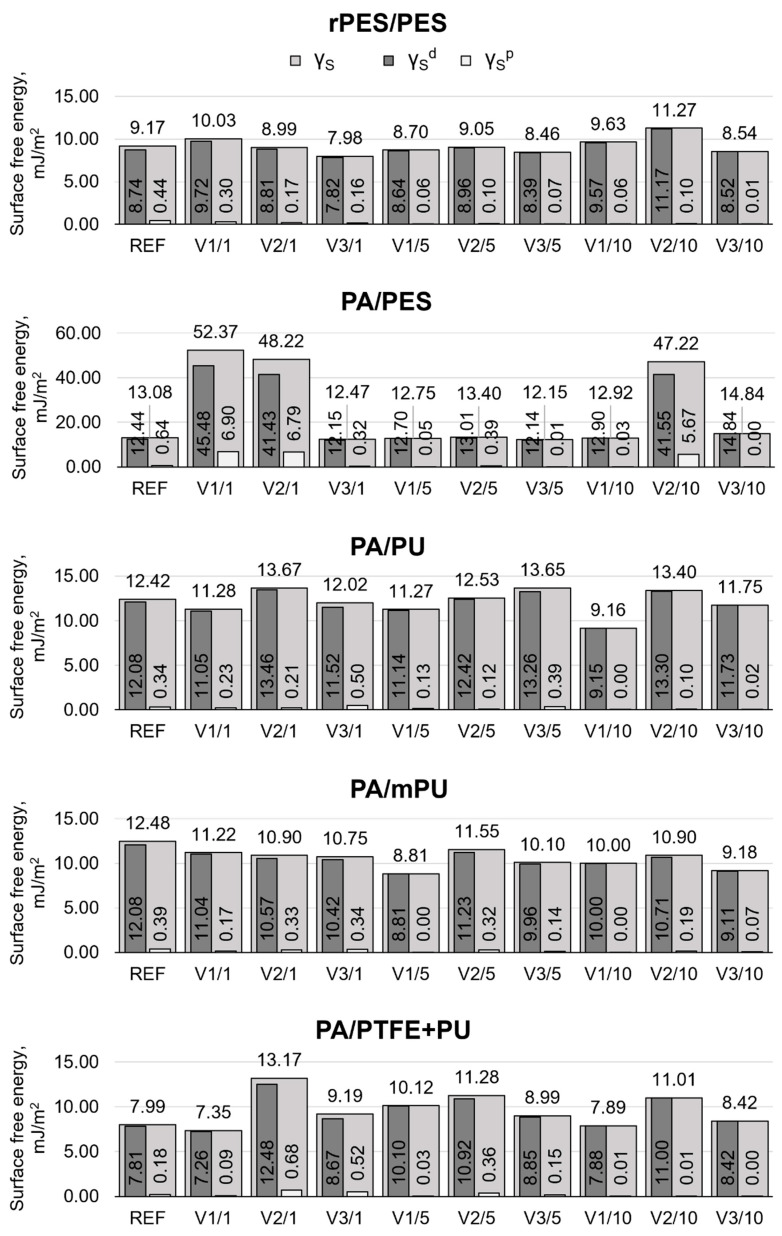
The average values of surface free energy (γ_S_) and its components, γ_S_^d^ and γ_S_^p^.

The study also showed the influence of the washing processes on the values of the surface free energy of the materials (including the dispersion and polar components), especially at those stages during which the average values of the contact angles significantly differed from the REF samples. Figure 7 presents the results of the surface free energy.

The values of surface free energy for the REF samples ranged from 7.99 mJ/m^2^ to 13.08 mJ/m^2^ (Figure 7). The lowest value of this parameter was recorded for the PA/PTFE + PU material with the highest fluorine content (5.13 ± 0.36%). Fluorine is an electronegative element, which means that it has a low affinity for electrons of other substances, including water. The contribution of the dispersion component to the total SFE of the PA/PTFE + PU material was over 97% (7.81 mJ/m^2^). The highest value of the surface free energy in the blank sample was recorded for PA/PES material with a PFC-free finish. The correlation analysis between the fluorine content of the REF samples and the corresponding free surface energy values showed a very high negative correlation (r = −0.83). The Pearson correlation coefficient indicates that the higher the percentage of fluorine on the surface of the materials, the lower the value of the surface free energy and, consequently, the lower the water wettability.

Washing caused changes in the mean values of surface free energy, the nature and magnitude of which depended on the variant and frequency of washing. For materials with a fluorine-based hydrophobic finish, a decrease in mean SFE values, compared to the REF sample, was observed after home washing with an all-purpose laundry detergent (V3), as well as after washing under standardized conditions (V1), after each washing multiplicity. Based on the analysis of significance of differences (Table 9), it was found that only after 10 washings, according to variants V1 and V3, were the values of the surface free energy significantly lower, compared to the SEP values for the REF samples (*p* < 0.05). The exception are the values of surface free energy of the PA/mPU material (Figure 7), for which, a decrease, in comparison with the REF samples, was observed for each washing multiplicity. The largest change for PA/mPU was found after five washes, according to V1 (−29%).

A statistically significant increase in the mean SFE values, compared with the REF samples, was observed after one and ten washings, according to V2 (Table 9). It amounted, on average, to 1.15 mJ/m^2^ (+11%, compared to the REF sample). The highest increase in surface free energy values among the materials with PFC finish (Figure 7) was observed for the PA/PTFE + PU material after one and ten washings, according to V2, by 65% and 38%, respectively. The highest increase in SFE among all materials after washing was observed for the PA/PES material with PFC-free finish, whose surface free energy value after one washing, according to V1, increased by 39.29 mJ/m^2^ (+300%, compared to the REF sample). An equally large increase in SFE for this material was determined at stages V2/1 (+269%) and V2/10 (+261%). 

Upon analyzing the data (Figure 5 and Figure 7), in terms of the effect of the washing conditions, it was found that washing according to the variant V2, in which a specialized detergent designed for rainwear (D2) was used, caused the largest (statistically significant) changes in the mean values of the contact angles for water and surface free energy, so its effect on the surface properties of the tested materials can be considered the most unfavorable. According to Davies [21], the deterioration of the hydrophobic properties of the materials with fluorine-based hydrophobic finishes, due to washing processes, may be caused by changes in the orientation of fluoroalkyl groups, as well as by the adsorption of detergents that increase the susceptibility of textiles to wetting. Surfactant residues may have contributed to the increased affinity for polar liquids (water).

## 4. Conclusions

The results of the research have shown that washing water-repellent materials with hydrophobic finishes can cause significant changes in their properties. The magnitude and character of these changes depends on the composition and structure of the material, type of hydrophobic finish, and washing conditions and frequency. 

The material with the PFC-free finish is the least resistant to washing. For all materials with PFC finishes, the value of the surface free energy is lower and depends not only on the fluorine content on the surface but also on the fabric topography, as evidenced by the values of the dispersion and polar components. 

The increase in the washing number resulted in a decrease in water repellency. The loss of water repellency below an acceptable level (Grade 3) occurred for all materials after the fifth washing. The values of the contact angle θ_W_ also decreased after washings, but they remained at a high level (above 120°). 

The statistical analysis of the spray test results did not show the significance of the effect of the washing variant on the durability of the water-repellent properties; however, the highest average decrease in the water repellency was observed for the V2 variant with the detergent D2 dedicated to the multifunctional clothing.

A novel approach to assessing the water-repellent properties of textile materials by using both the spray test method and contact angle measurements showed the differences in the evaluation and interpretation of the water-repellent properties. The spray test method shows how the clothing material will behave in real conditions, while the contact angle measurements and determination of the surface free energy make it possible to identify the causes and determine the direction of changes in the properties of water-repellent materials. It was shown that high values of contact angles obtained for materials after washing do not necessarily prove the high effectiveness of water-repellent finishes, which was confirmed by the results of the spray test. Therefore, we recommend using both methods (i.e., the spray test and goniometric method) to assess the changes in the water-repellent properties of textile materials, which allows for obtaining more information on materials with water-repellent finishes. 

The results of our study indicate the possibilities for the deeper exploration of this issue. Future research in this area may focus on examining the effects of material composition, structural parameters, and 3D surface topography, as well as more diverse detergent types and maintenance conditions (including drying), on the durability of water-repellent finishes.

## Figures and Tables

**Table 1:** Characteristics of laminated textile materials provided by producers.

Material Symbol	Structure	Composition		DWR Finishing Type
		Fabric	Membrane (Type)	
rPES/PES	2-layer	recycled polyester	polyester (hydrophilic)	PFC
PA/PES	2-layer	polyamide 6.6	polyester (hydrophilic)	PFC-free
PA/PU	2-layer	polyamide 6.6	polyurethane (hydrophilic)	n/d
PA/mPU	2-layer	polyamide 6.6	polyurethane (microporous)	n/d
PA/PTFE + PU	2.5-layer	polyamide 6.6	polytetrafluoroethylene (microporous with additional hydrophilic polyurethane coating)	n/d

**Table 2:** Structural parameters of laminated textile materials used in the study.

Material Symbol	Thickness [mm]	Mass per Unit Area [g/m^2^]	Number of Threads per 1 cm		Type of Fabric Weave
			ISO 7211-2		
	ISO 5084	EN 12127	Warp	Weft	ISO 3572
rPES/PES	0.46 ± 0.00	211 ± 1	22 ± 1	19 ± 0	plain
PA/PES	0.19 ± 0.00	175 ± 0	55 ± 0	53 ± 1	plain
PA/PU	0.32 ± 0.00	179 ± 2	55 ± 1	46 ± 0	plain
PA/mPU	0.36 ± 0.00	193 ± 1	55 ± 0	49 ± 1	plain
PA/PTFE + PU	0.26 ± 0.01	193 ± 1	44 ± 1	33 ± 0	twill (1/2 S)

**Table 3:** List of test samples.

Symbol	Samples
REF	Reference sample (before washing)
V1/1; 5; 10	Fabric after 1, 5, and 10 washes in detergent D1
V2/1; 5; 10	Fabric after 1, 5, and 10 washes in detergent D2
V3/1; 5; 10	Fabric after 1, 5, and 10 washes in detergent D3

**Table 4:** Resistance to surface wetting (spray test) rating.

ISO 4920	AATCC TM22	Characteristic
5	100	No sticking or wetting of the specimen face
4	90	Slight random sticking or wetting of the specimen face
3	80	Wetting of the specimen face at spray points
2	70	Partial wetting of the specimen face, beyond the spray points
1	50	Complete wetting of the entire specimen face, beyond the spray points
–	0	Complete wetting of the entire face of the specimen

Source: ISO 4920, AATCC TM22.

**Table 5:** Characteristic of standard liquids.

Standard Liquid	Surface Tension, mJ/m^2^				
	γ	γ^d^	γ^p^	γ^+^	γ^−^
Water (distilled)	72.8	21.8	51.0	25.5	25.5
Diiodomethane	50.8	50.8	0	0	0

**Table 6:** Results of EDS analysis of fabric surfaces.

Element	Mean Weight Percentage of Elements [%]				
	rPES/PES	PA/PES	PA/PU	PA/mPU	PA/PTFE + PU
C	60.91 ± 0.06	65.27 ± 0.27	64.69 ± 0.52	64.65 ± 0.26	64.20 ± 0.23
O	36.38 ± 0.28	20.20 ± 0.06	19.22 ± 0.16	19.45 ± 0.09	17.56 ± 0.09
N	–	13.46 ± 0.27	13.36 ± 0.66	13.77 ± 0.20	11.64 ± 0.35
F	2.15 ± 0.33	–	2.31 ± 0.03	1.49 ± 0.13	5.13 ± 0.36
Ti	0.43 ± 0.01	0.84 ± 0.01	0.15 ± 0.01	0.16 ± 0.02	0.91 ± 0.04
Cl	0.13 ± 0.01	–	0.09 ± 0.01	0.15 ± 0.01	0.42 ± 0.02
S	–	0.11 ± 0.01	0.18 ± 0.01	0.19 ± 0.01	0.16 ± 0.01
Si	–	0.11 ± 0.01	–	0.14 ± 0.01	–
Total	100.00	100.00	100.00	100.00	100.00

**Table 7:** The results of the analysis variance (ANOVA) of the influence of the washing variant on the resistance to surface wetting.

	Variant 1	Variant 2	Variant 3	*p*-Value
Mean ± SD	−1.75 ± 1.22	−2.76 ± 1.18	−1.94 ± 1.59	0.064
Lower quartile (Q1)	−2.79	−3.62	−2.79	
Median (Q2)	−1.92	−3.25	−1.83	
Upper quartile (Q3)	−0.62	−2.54	−0.46	

**Table 8:** The average values of water (θ_W_) and diiodomethane (θ_DIM_) contact angles and their standard deviation.

Sample	rPES/PES		PA/PES		PA/PU		PA/mPU		PA/PTFE + PU	
	θ_W_, Deg	θ_DIM_, Deg	θ_W_, Deg	θ_DIM_, Deg	θ_W_, Deg	θ_DIM_, Deg	θ_W_, Deg	θ_DIM_, Deg	θ_W_, Deg	θ_DIM_, Deg
REF	138.7 ± 1.2	102.2 ± 1.0	134.8 ± 0.8	93.4 ± 0.8	132.0 ± 1.9	93.6 ± 1.6	132.7 ± 1.7	93.7 ± 1.0	136.5 ± 1.0	104.2 ± 1.0
V1/1	135.1 ± 0.7	99.3 ± 0.9	130.6 ± 2.4	41.4 ± 2.0	131.9 ± 0.9	95.8 ± 1.2	130.9 ± 1.6	95.6 ± 1.3	135.4 ± 1.1	105.5 ± 1.2
V2/1	134.5 ± 1.3	101.3 ± 1.1	133.3 ± 2.4	48.2 ± 0.9	128.3 ± 0.8	90.3 ± 0.9	134.0 ± 1.6	97.2 ± 1.3	135.2 ± 0.8	93.3 ± 1.0
V3/1	136.1 ± 1.1	104.1 ± 1.2	131.6 ± 1.2	93.4 ± 1.3	134.7 ± 1.7	95.3 ± 0.7	134.4 ± 1.1	97.6 ± 1.2	139.8 ± 1.2	102.5 ± 1.4
V1/5	132.1 ± 1.3	101.3 ± 1.5	125.8 ± 0.7	91.3 ± 1.1	130.0 ± 1.8	95.3 ± 2.2	128.9 ± 0.9	100.4 ± 0.8	128.7 ± 1.3	97.3 ± 1.2
V2/5	132.7 ± 1.0	100.7 ± 0.8	131.3 ± 1.1	91.7 ± 1.0	127.9 ± 0.7	92.2 ± 1.4	133.0 ± 0.8	95.6 ± 0.8	133.9 ± 1.3	96.4 ± 0.5
V3/5	132.8 ± 1.2	102.1 ± 0.9	122.3 ± 0.7	91.7 ± 0.8	130.9 ± 0.9	91.1 ± 0.8	132.0 ± 1.3	98.2 ± 1.3	133.9 ± 0.8	101.1 ± 1.1
V1/10	130.7 ± 1.1	98.9 ± 1.1	124.9 ± 1.5	90.7 ± 1.0	127.2 ± 1.1	99.2 ± 1.1	126.9 ± 1.1	97.2 ± 1.1	128.2 ± 1.1	102.6 ± 1.2
V2/10	129.3 ± 1.2	95.1 ± 1.2	129.8 ± 0.8	47.2 ± 1.5	126.4 ± 1.2	90.2 ± 1.1	131.7 ± 1.3	96.5 ± 1.2	124.0 ± 0.9	94.5 ± 1.2
V3/10	130.5 ± 0.9	101.3 ± 1.1	120.2 ± 0.8	86.1 ± 1.3	126.0 ± 1.1	93.3 ± 0.9	131.6 ± 1.3	100.1 ± 0.6	128.2 ± 1.1	101.2 ± 0.7

**Table 9:** Analysis of statistical significance (*p*-value) of the changes in water and diiodomethane contact angles, surface free energy values, and components, based on Student’s *t*-test (N = 20).

Sample	Water Contact Angle, Deg		Diiodomethane Contact Angle, Deg		Surface Free Energy, mJ/m^2^		Dispersive Component, mJ/m^2^		Polar Component, mJ/m^2^	
	Mean ± SD	*p*-Value	Mean ± SD	*p*-Value	Mean ± SD	*p*-Value	Mean ± SD	*p*-Value	Mean ± SD	*p*-Value
REF	135.0 ± 3.2	–	98.4 ± 5.5	–	10.51 ± 2.28	–	10.18 ± 2.23	–	0.34 ± 0.11	–
V1/1	133.3 ± 2.2	0.033	99.0 ± 4.6	0.345	9.97 ± 1.84	0.188	9.77 ± 1.78	0.248	0.20 ± 0.09	0.001
V2/1	133.0 ± 3.2	0.026	95.5 ± 4.8	0.029	11.68 ± 2.16	0.041	11.33 ± 2.06	0.036	0.35 ± 0.23	0.468
V3/1	136.2 ± 2.5	0.081	99.9 ± 4.1	0.162	9.98 ± 1.77	0.190	9.61 ± 1.67	0.163	0.38 ± 0.17	0.257
V1/5	129.9 ± 1.6	<0.001	98.6 ± 2.8	0.460	9.73 ± 1.22	0.087	9.67 ± 1.18	0.179	0.05 ± 0.06	<0.001
V2/5	131.9 ± 2.7	<0.001	96.2 ± 3.5	0.052	11.10 ± 1.47	0.154	10.88 ± 1.44	0.103	0.22 ± 0.14	0.006
V3/5	132.4 ± 1.3	0.001	98.1 ± 5.0	0.418	10.30 ± 2.33	0.371	10.11 ± 2.20	0.462	0.18 ± 0.14	0.001
V1/10	128.3 ± 1.7	<0.001	99.5 ± 2.3	0.206	9.17 ± 0.92	0.006	9.15 ± 0.92	0.024	0.02 ± 0.03	<0.001
V2/10	127.9 ± 3.3	<0.001	94.1 ± 2.7	<0.001	11.64 ± 1.18	0.022	11.55 ± 1.18	0.007	0.10 ± 0.07	<0.001
V3/10	129.1 ± 2.5	<0.001	99.0 ± 3.8	0.348	9.47 ± 1.56	0.037	9.45 ± 1.56	0.099	0.03 ± 0.03	<0.001

## Data Availability

Not applicable.
